# Chromosomal rearrangements in acute myeloid leukemia (AML)

**DOI:** 10.3332/ecancer.2010.183

**Published:** 2010-09-30

**Authors:** E Belloni, M Trubia, P Gasparini, C Micucci, C Tapinassi, S Confalonieri, P Nuciforo, B Martino, F Lo-Coco, PP Di Fiore, PG Pelicci

**Affiliations:** 1 Istituto Europeo di Oncologia, Milan, Italy; 2 Campus IFOM-IEO, Milan, Italy; 3 Divisione di Ematologia, Azienda Ospedaliera Bianchi-Malacrino-Morelli, Reggio Calabria, Italy; 4 Università di Roma Tor Vergata, Department of Biopatologia e Diagnostica per Immagini, Rome, Italy; 5 Dipartimento di Medicina, Chirurgia ed Odontoiatria, Universita’ degli Studi di Milano, Milan, Italy

**Figure:**
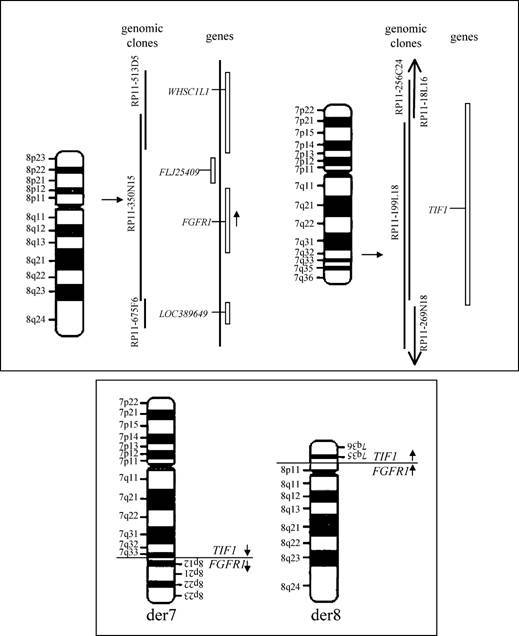


**Figure:**
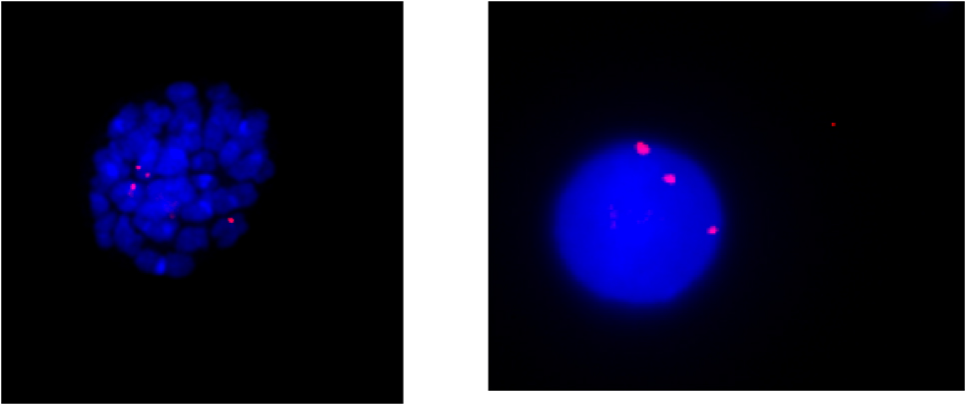


Chromosomal rearrangements in acute myeloid leukaemia (AML) frequently give rise to aberrant fusion proteins, responsible for the disease onset (50% of all reported cases). Five per cent of such cases harbour rare rearrangements, mostly translocations, resulting either in new rare fusion products or in the deregulation/truncation of specific genes. In this specific case, a new rearrangement between chromosomes 7 and 8 gives rise to a new abnormal fusion product between the *FGFR1* and *TIF1* genes.

Fluorescence *in situ* hybridization (FISH) can be used to evidence the presence of such a rearrangement, by selecting genomic clones mapping in the chromosomal region that contains the breakpoint. The presence of the rearrangement can be detected in both interphase and metaphase nuclei. In the shown image, the clone 350N11, spanning the FGFR1 locus, has been labelled in red and used to hybridize interphase and metaphase nuclei (stained with DAPI, blue) on bone marrow cells derived from the patient. The presence of three signals indicates the clone is recognizing the normal chromosome 8, as well as the two derivative chromosomes 7 and 8, proving the presence of the breakpoint within this gene.

